# Panoptic Overview of Triple-Negative Breast Cancer in Nigeria: Current Challenges and Promising Global Initiatives

**DOI:** 10.1200/JGO.17.00116

**Published:** 2018-06-04

**Authors:** Nikita Wright, Padmashree Rida, Emad Rakha, Ayodeji Agboola, Ritu Aneja

**Affiliations:** **Nikita Wright**, **Padmashree Rida**, **Emad Rakha**, **Ayodeji Agboola**, and **Ritu Aneja**, Georgia State University, Atlanta, GA; **Emad Rakha**, University of Nottingham and Nottingham University Hospitals National Health Service Trust, Nottingham, United Kingdom; and **Ayodeji Agboola**, Olabisi Onabanjo University, Sagamu, Nigeria.

## Abstract

**Purpose:**

Triple-negative breast cancer (TNBC) is the most deadly form of breast cancer (BC) today. TNBC treatment is fraught with challenges because of the extensive interpatient heterogeneity in clinical behavior and scarcity of stratifying biomarkers and actionable targets. Women of African ancestry face a disproportionate burden resulting from this disease, which affects them earlier and more aggressively and has a higher propensity to spread and resist conventional treatments. A much higher proportion of Nigerian patients with BC have TNBC compared with patients with BC in the United States and Europe.

**Methods:**

This article spotlights Nigeria as an example of a nation wherein genetic and nongenetic spheres of influence intersect to affect the prevalence of this disease, the scale of its challenge, and its toll.

**Results:**

Studies have illuminated the inherently different tumor biology of Nigerian TNBCs, which show distinct genetic variants and gene expression patterns compared with European or European-American TNBCs. Parallels are apparent between TNBC phenotypes among African Americans and Nigerians, implicating the common thread of shared genetic ancestry between these populations. Reproductive, lifestyle, socioeconomic, and cultural factors also shape TNBC outcomes in Nigeria, as do resource constraints in Nigerian health care and research sectors.

**Conclusion:**

Increasing our understanding of how these factors contribute to poorer outcomes among Nigerian women may uncover valuable insights and strategies in alleviating the TNBC burden in many countries of the world and help reduce the racial disparity in BC-related outcomes here in the United States. Importantly, this review also highlights collaborative global and local initiatives that converge expertise and resources to advance research on effective management of TNBC in diverse populations.

## TRIPLE-NEGATIVE BREAST CANCER: A CONFLUENCE OF COMPLEX CHALLENGES

Triple-negative breast cancer (TNBC) accounts for 20% (approximately 0.17 million) of breast cancer (BC) cases worldwide but remains the most deadly subgroup of BCs.^1^ Defined by the absence of therapeutically targetable estrogen receptor (ER), progesterone receptor, and human epidermal growth factor receptor 2 overexpression, TNBCs often present with more aggressive clinicopathologic features (eg, basal-like phenotype, higher grade and stage, greater proliferation) than luminal tumors.^1,2^ Currently, no targeted therapies are approved for TNBC; thus, surgery, anthracycline- and taxane-based chemotherapy, and radiation therapy are the primary treatment options for patients with TNBC. Despite these treatments, TNBCs run a high risk of progression, especially within the first 5 years after diagnosis.^3^ Rampant interpatient and intratumor heterogeneity render management of this disease complex and warrant deeper dissection of the molecular landscape of TNBC.^4^ Nongenetic factors such as health care facilities, resource constraints in patients’ countries, and lifestyle, epidemiologic, and cultural factors all collude and contribute to the burden that TNBC imposes on patients and families, as the disease takes away years from life and life from years. Thus, enhancing our understanding of tumor biology and modifiable factors that influence clinical outcomes, identifying better biomarkers for patient stratification, and developing newer, more effective targeted therapies are critical for improving TNBC management globally.

Several studies have suggested that biogeographic ancestry might be a key driver of aggressive BC. Women of African ancestry are disproportionately affected by TNBC, with poorer clinical outcomes compared with patients with BC of other ethnicities. In the United States, African American (AA) women are two to three times more likely to be diagnosed with TNBC compared with European Americans (EAs).^5^ Among patients with TNBC, AAs are more likely to experience rapid disease progression and shorter survival times than EAs. Furthermore, TNBC is significantly more prevalent and presents with higher grade and earlier onset among West African (WA) women compared with AA women.^6^ Thus, clearer understanding of the interplay between genetic and nongenetic causes of higher TNBC prevalence in WA women, who share a common ancestry with AAs because of the trans-Atlantic colonial slave trade, may allow us to design better strategies (health care guidelines and policies, preventative measures, strategic investment in infrastructure) to ameliorate global TNBC burden and racial disparity in BC outcomes in the United States. Essentially, this review aims to dissect the complex landscape of Nigerian TNBC to understand drivers of the disproportionate burden of TNBC in WA women and racial disparity in outcomes in the United States and to highlight concerted global and local initiatives and collaborative interventions across multiple stakeholders that aim to drive sustainable change and reduce the devastating footprint of this disease.

## RETURNING TO OUR ROOTS: DIGGING DEEP INTO THE LANDSCAPE OF TNBC IN NIGERIA TO BETTER MANAGE TNBC WORLDWIDE

“The roots of education are bitter, but the fruit is sweet.” —Aristotle

Ancestry genotyping studies show that the AA population predominantly harbors West or West-Central African ancestry, presumably because of the colonial slave trade.^7^ Mortality rates have been reported to be much lower among East African compared with WA patients with BC, suggesting that WA ancestry may explain, at least in part, poorer clinical outcomes between AA and EA patients.^8^ Nigeria, the largest and most populous developing country in Africa, exemplifies the current dismal state of BC in WA. There are more than 27,000 new cases of BC annually in Nigeria; roughly 70% to 80% of these patients present with locally advanced or metastatic BC, and nine of 10 of these women die within the first 5 years.^9^ The mean age at presentation for Nigerian patients with BC is approximately 43 years, with 74% identified as premenopausal and 12% younger than age 30 years.^10^ A large percentage of Nigerian BC cases are often classified as TNBC, although it has been suggested that many of these cases are false negatives, resulting from suboptimal tissue fixation and pathology practices.^11,12^ Over the next few sections, we discuss contributions of genetic and nongenetic factors to the etiology and prognosis of TNBC in Nigeria and explore how this knowledge could inform clinicians about strategies to more effectively overcome the disease.

## WE CANNOT HELP WHAT WE ARE BORN WITH: GENETIC RISK FACTORS IN NIGERIAN TNBC

“We can’t choose where we come from, but we can choose where we go from there.” —Steven Chbosky

The stark disparity in TNBC prevalence and mortality between WA women and women of other ancestral backgrounds has spurred efforts to elucidate its genetic basis, as summarized in Table 1. Studies have identified an association of African ancestry as well as genetic variants, more prevalent among women of African descent, with increased risk for more aggressive BC phenotypes and advanced stage.^13,14^ Mutations in tumor suppressor genes such as *BRCA1* have also been implicated in the higher TNBC incidence rate in Nigeria.^15,16^ Furthermore, studies have uncovered multiple biomarkers that are generally overexpressed in Nigerian and WA patients with BC and are associated with TNBC status and aggressive clinicopathologic characteristics (Table 1).^17-24^ However, additional research is necessary to uncover selectively targetable inherent tumor biologic characteristics in Nigerian women to improve their disease outcomes. In the United States, the AA population is highly admixed and typically harbors 14% to 21% European ancestry and approximately 1% to 3% Native American ancestry.^25^ Thus, ancestry genotyping, which has hitherto received scant consideration, has to be more firmly embedded in biomarker and drug discovery and development studies and clinical trial designs so that the personal can be more fully reinstated in personalized medicine for TNBC.

**Table 1:**
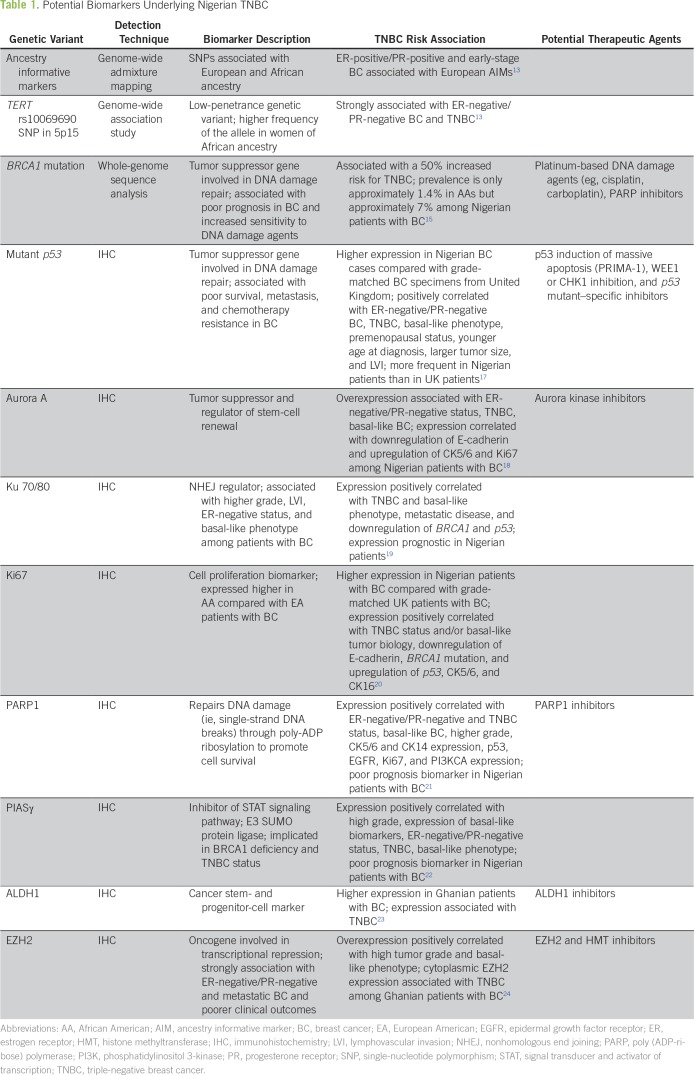
Potential Biomarkers Underlying Nigerian TNBC

## NATURE VERSUS NURTURE: WHAT WE KNOW AND NEED TO KNOW ABOUT NONGENETIC RISK FACTORS FOR TNBC IN NIGERIA

“You inherit your environment as much as your genes.” —Johnny Rich

Studies are increasingly showing that behavioral and social factors profoundly influence disease risk, and TNBC is no exception. Because these are modifiable via appropriate interventions, their due consideration via a life course approach could affect TNBC diagnoses, prevalence rates, management strategies, and outcomes in Nigeria.

### Epidemiologic Risk Factors

#### Reproductive factors.

A positive association between higher parity and increased likelihood of developing TNBC and a negative association between breastfeeding duration and risk of developing TNBC have been detected in AA women.^26,27^ Furthermore, one study reported that the use of oral hormonal contraceptives is associated with a 2.9 times greater risk of TNBC in women age between 45 and 64 years.^28^ Thus, these associations warrant further study in the Nigerian population.

#### Anthropometric factors.

Obesity has been linked to increased risk for developing TNBC in women of all ethnic backgrounds.^29^ Obesity is increasing in Nigeria because of increased consumption of calorie-dense foods.^30^ A high hip-to-waist ratio, more prevalent among women of African descent, has been linked to increased risk for developing TNBC or ER-negative/progesterone receptor–negative BC in women of African descent.^27^ Furthermore, a study conducted in Nigeria reported that height is significantly linked to increased risk for developing BC; thus, it may be interesting to investigate if height is associated with increased risk of TNBC in Nigeria.^31^

#### Lifestyle.

Diet and nutrition may play a critical role, according to one study, which found that there is an inverse association between a diet high in fruits and vegetables and risk of developing ER-negative BC.^32-34^ Alcohol consumption, smoking, and physical inactivity have all been linked to increased BC risk in Nigeria; thus, it may be worthwhile to investigate their effects on TNBC risk in Nigeria.^35-37^

#### Socioeconomic status.

Irrespective of ancestral background, living in poor socioeconomic conditions can increase a woman’s chances of being diagnosed with TNBC over other BC subtypes.^38^ Nigeria ranks among the poorest nations in the world, with a population of more than 150 million but a gross domestic product of only US$2,000 per capita annually.^39^ A study conducted in a Nigerian tertiary hospital found that almost 45% of the patients declined treatments in the middle of their chemotherapy regimens because of financial instability.^39^ Thus, the poor, marginalized, and rural women of the country bear the most acute brunt of the burden of TNBC.

#### Health literacy and education.

A clinical study conducted at the University College Hospital in Ibadan, Nigeria, found that approximately 85% of Nigerian patients with BC presented at an advanced stage.^40^ Lack of knowledge of BC signs and symptoms are serious barriers to Nigerian patients receiving timely and adequate treatment. Although some studies report approximately 80% to 92% of Nigerian women are aware of mammography screening, only 3% to 10% are reported to have actually undergone the screening.^41^ Studies have revealed that Nigerian women with a higher level of education exhibit more knowledge about breast self-examination (BSE), tend to believe that early BC detection leads to better survival rates, and are more likely to practice BSE compared with those with a lower level of education.^42-44^ Studies have also revealed that most Nigerian health care providers lack sufficient knowledge of BC risk factors and the procedure for BSE^.^^4^^,^^5^ Also, single marital status, premenopausal status, fear of discovering a lump, and residing in rural or remote areas have been associated with delay in seeking medical attention in Nigeria.^41,45-49^ A majority of Nigerian women obtain their BC information from television (31%), clinics (31%), and health professionals as well as from their elders, friends, and neighbors among rural women according to one study; thus, disparities in socioeconomic status may underlie differences in awareness and stage at presentation between semiurban and rural Nigerian communities.^48,50-52^ These studies highlight a need for continuing medical and health education programs to improve awareness among the Nigerian adult population and health care professionals of early detection to improve outcomes. Several nonprofit organizations or nongovernmental societies and community outreach programs have been established in Nigeria to improve BC awareness to eventually reduce societal, financial, emotional, and health burdens resulting from TNBC.

### Cultural Norms and Beliefs

Cultural traditions and spiritual beliefs in Nigeria can influence a patient’s decision to seek timely medical treatment. Some Nigerian women believe that unless the swelling or lump in the breast is painful, it is unlikely to be malignant, and they do not need medical attention.^53^ Another study found that 17.5% of Nigerian patients with BC initially consulted with traditional healers for treatment, which was associated with a more than 3-month delay until presentation.^54^ Also, in some African cultures, people believe that BC is caused by social misconduct, such as oral or nipple contact, or a woman wearing unclean garments.^55^ Fear of being divorced by her husband or of being ostracized by the community, fear of disfigurement by surgery, fear of pain or embarrassment during medical examinations (especially if the medical practitioner is of the opposite sex), fear of ineffective treatment, lack of confidence in physicians, belief that surgery accelerates metastasis, fear of death, and other factors, including lack of family support, may also deter a woman from seeking medical help immediately.^55^ Thus, to reduce the TNBC burden in Nigeria, there is a critical unmet need to develop a more nuanced understanding of the wider social context of the human lives it affects and develop focused interventions to address all exacerbating factors.

### Other Potential Risk Factors

Infectious agents or environmental estrogens such as insecticides and dichlorodiphenyltrichloroethane, used for preventing insect-borne diseases like malaria, are postulated to elevate risk for developing hormonal-related diseases, because they can alter hormone levels.^43^ Cosmetic products frequently used among African women, such as hair relaxers and skin lighteners, may also be contributing to increased risk for TNBC among Nigerian women, because these contain dangerous carcinogens and/or hormonally active compounds.^43^ Their potential influence on TNBC risk in Nigeria merits further study.

## ADDING FUEL TO THE FIRE: CHALLENGES IN TNBC CARE AND TREATMENT IN NIGERIA

“Access to healthcare shouldn’t depend on your postcode.” —Richard Di Natale

The resource-constrained Nigerian health care system, lack of education and empowerment, poverty, shortage of well-trained health care personnel, and inadequate research infrastructure, as compared with other countries in the world such as the United States, have had significant adverse impacts on TNBC outcomes among the Nigerian population.

### Inadequate Health Care Infrastructure

A study conducted in a Nigerian teaching hospital in southwestern Nigeria found that the average duration between onset of BC symptoms and presentation was approximately 11.2 months, and approximately 39% of women presented with fungating tumors.^43^ In addition to aforementioned cultural and awareness-related factors, the delay in seeking medical attention may also be attributed to prohibitive cost of treatment, lack of transportation or access to radiology and chemotherapy facilities, and hospital overcrowding.^56-60^ In Nigeria, out-of-pocket expenditure for health care was an alarming 95.7% in 2014 according to the WHO. WHO reported that Nigeria allocates only $67 per person for health care, and financial constraints were the primary reason for patients with BC discontinuing treatments. Quality of health care also leaves much to be desired, and inappropriate surgeries and biopsy management lead to late-stage presentation.^61^ Palliative care is often the only option for patients diagnosed in advanced stages; however, pain medications are often limited or unavailable, especially in rural areas.^62^

Inferior health care infrastructure, especially paucity of facilities for BC detection and treatment, majorly underlies late presentation and poor clinical outcomes among Nigerian patients with TNBC. Nigeria is one of the least developed countries with regard to oncology services, resources, and radiation therapy facilities.^9^ Nigeria houses seven radiotherapy laboratories; however, only 15% of the reported 4 million Nigerians needing radiotherapy have access to these facilities.^63,64^ Furthermore, lack of trained personnel to properly operate radiotherapy equipment results in poor maintenance and equipment malfunction.^9^ The majority of the radiology equipment at cancer treatment centers is considered nonfunctional because of a lack of qualified personnel who can properly handle the equipment.^65^ The frequent breakdown of radiotherapy equipment may be attributed to equipment procurement without consultation or advice from end users, unreliable electricity, high cost of operation, bottlenecks in securing spare parts, absence of maintenance contracts with suppliers, and lack of quick response from foreign engineers when equipment malfunctions.^9^ In addition, mammography facilities are scarce in Africa, and they inadequately detect cancer in women with dense breasts.^66^ Mammography screening may not detect all tumors in women at the premenopausal age, when the bulkiness of the breast is often an interference in Nigerian women.^11,67^ Furthermore, public hospitals are often overcrowded, lack human resources, and require long waiting periods, which stalls screening, diagnosis, and treatment of patients.^68^ Also, lack of follow-up and poor patient recordkeeping in clinics interfere with determination of specific factors influencing survival patterns for refinement of treatment plans.^39^

#### Pathology practices.

Subpar pathology practices are prevalent in many centers in Nigeria and often result in inaccurate diagnoses and consequently inappropriate treatment. Incorrect immunohistochemistry results attributable to poor tissue collection or processing, delayed fixation or overfixation, poor-quality reagents, incorrect laboratory techniques, and lack of quality assurance practices are often prime suspects. Records about cause of death are often not notifiable or centrally maintained. Although Nigeria is one of the few African countries that have published guidelines, created by the Nigeria Breast Pathology Working Group in 2010, on standardized pathology reporting, these guidelines have not been adequately circulated or implemented in medical centers. Daramola et al^69^ compared histologic parameters in pathology reports from a teaching hospital in Nigeria with the cancer data set of the Royal College of Pathologists in the United Kingdom to verify compliance and concordance. Almost half of the Nigerian BC cases examined were discordant with the Royal College of Pathologists, and roughly half of the cases were either undergraded or overgraded. Poor fixation and exclusion of mitotic count were underlying factors for discordant grading. Thus, proper training and education of Nigerian pathologists are critical to improving accuracy in BC diagnosis and reporting. Templates or proformas have been suggested for use by African pathologists to guide accurate reporting.

#### Lack of health care personnel.

The severe shortage of competent health care providers, including oncologists, radiologists, surgeons, and pathologists, has also contributed to the poor BC-related outcomes in Nigeria. The number of physicians per 100,000 people in Africa is presently 12, which is much lower than the 387 physicians per 100,000 people in Europe.^70,71^ The number of physicians per 100,000 people in Nigeria is currently 18.8, which is much lower than the numbers per 100,000 people in most Western nations, including the United States, where there are approximately 148 physicians per 100,000 people.^71,72^ Nigeria currently ranks seventh highest among 57 countries in the world facing a health care shortage crisis, according to the Federal Ministry of Health. Adebayo et al^72^ reported that there are 33.% and 29.3% gaps in the supply of doctors and nurses, respectively, in Nigeria.^72^ The shortage of pathologists in Nigeria is also extreme, with only 6% of practicing specialist physicians certified as pathologists.^73^ Furthermore, Nigeria possesses fewer than 40 trained radiation oncologists to provide radiology services and meet the needs of the growing population of patients with cancer.^65^ The paucity of practicing clinicians in African countries can be attributed to a significant reduction (6% to 18%) in medical school teaching staff, who have opted to emigrate over the past 5 years (phenomenon known as brain drain).^73,74^ Many trainees often leave countries in sub-Saharan Africa for developing countries because of inadequate infrastructure to practice, poor working conditions, and low remuneration.^73^ Implementing strategies to attract more African health care personnel, such as raising salaries and improving working conditions, will be crucial to ensuring adequate patient coverage. Fortunately, Nigeria possesses a number of highly skilled, motivated, and overseas-trained clinicians who can serve as conduits for revamping health care and conquering challenges of TNBC management.

### Research

Research provides the evidence base on which cancer prevention, control, and treatment strategies are built. The numbers of cancer researchers, including epidemiologists, statisticians, scientists, public health experts, health economists, and behavioral scientists, in Africa are limited. Although Nigeria is among the top countries in Africa for publishing research articles on BC, cutting-edge research is still lacking.^75^ There is also a significant shortage of cancer registries and trained cancer registry personnel. In 2006, only 11% of the African population was covered by a cancer registry.^76^ Nigeria has many population-based cancer registries, such as the Ibadan Cancer Registry located in South West Nigeria and the Abuja Cancer Registry located in North Central Nigeria.^77^ Both ensure high-quality cancer data; however, together they cover only 2.5% of the Nigerian population. In addition, the scarcity of data on cancer statistics and trends may be a result of government prioritization of funding of research on communicable diseases, rather than cancer research, infrastructure, or treatment. Furthermore, cancer advocates and trained community health care workers responsible for educating the public and policymakers on cancer are unable to meet the needs of the nation.

## TAKING MATTERS INTO OUR OWN HANDS: THE INTERNATIONAL CONSORTIUM FOR ADVANCING RESEARCH ON TNBC

“United we conquer, divided we fall.” —Aesop

**Fig 1:**
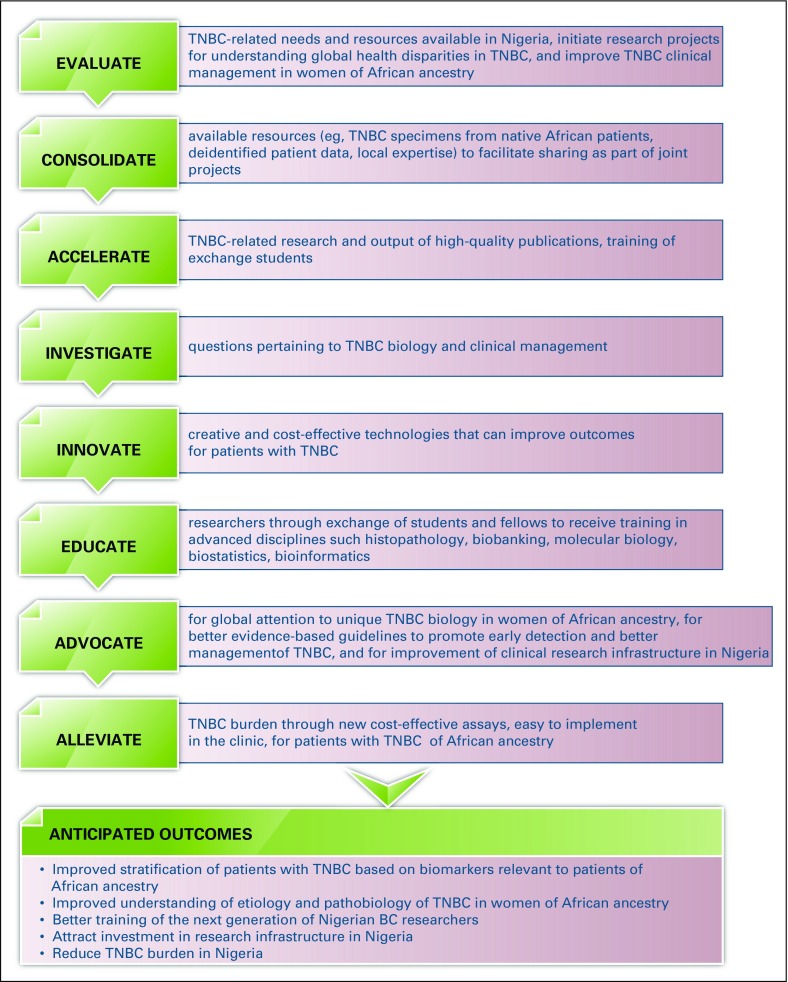
International Consortium for Advancing Research on Triple-Negative Breast Cancer (TNBC) strategic action plan for Nigeria. BC, breast cancer.

**Fig 2:**
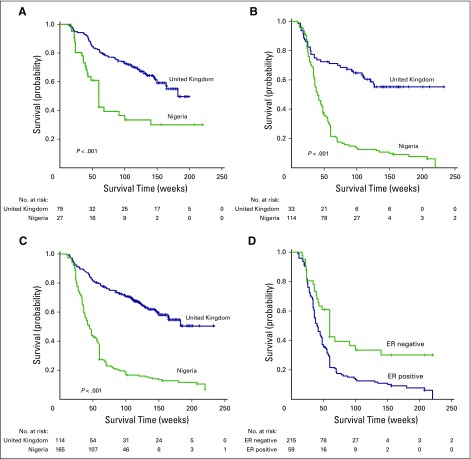
Overall survival comparison between patients with breast cancer (BC) in Nigeria and the United Kingdom. Overall survival of lymph node–matched patients with BC in Nigeria (n = 308) and the United Kingdom (n = 308) observed at Olabisi University Teaching Hospital in Sagamu, Nigeria, and the Nottingham-Tenuous Primary Breast Carcinoma Series, respectively, among (A) estrogen receptor (ER) –positive, (B) ER-negative, and (C) all cases. (D) Overall survival among Nigerian patients with ER-negative and ER-positive disease. Nigerian patients presented between January 2002 and December 2008, and UK patients presented between 1986 and 1993.

The increased global awareness of the alarming worldwide BC burden has sparked the launch of several global and local initiatives targeting BC and TNBC globally and in Nigeria (Appendix Table A1). However, global initiatives centered on alleviating the TNBC burden in Nigeria and around the world are almost nonexistent. In view of this unmet need to improve outcomes for patients with TNBC by invigorating multidisciplinary research on the fundamental biology of TNBC in diverse populations, our group, along with a leading BC research group led by Emad Rakha, MD, from Nottingham City Hospital (Nottingham, United Kingdom), jointly founded the International Consortium for Advancing Research on TNBC (ICART), a global coalition of TNBC researchers from the United States, Europe, Asia, and Africa. The mission of ICART is to consolidate, streamline, and share resources and mobilize and synergize complementary strengths to conduct large-scale multi-institutional and high-impact clinical, translational, and population-based research projects related to TNBC (Fig 1). ICART includes a network of 14 teaching hospitals across the length and breadth of Nigeria, and ICART researchers have already uncovered previously unrecognized disparities in breast clinicopathologic variables (Table 2), biomarker expression (Table 3), and survival (Fig 2) between lymph node–matched Nigerian and UK patients with BC that may underlie the stark disparity in clinical outcomes between these populations. ICART aims to leverage its network of highly skilled and well-qualified Nigerian clinicians and researchers to place Nigeria on the map for excellence in scientific research and health policy among low- to middle-income countries

**Table 2:**
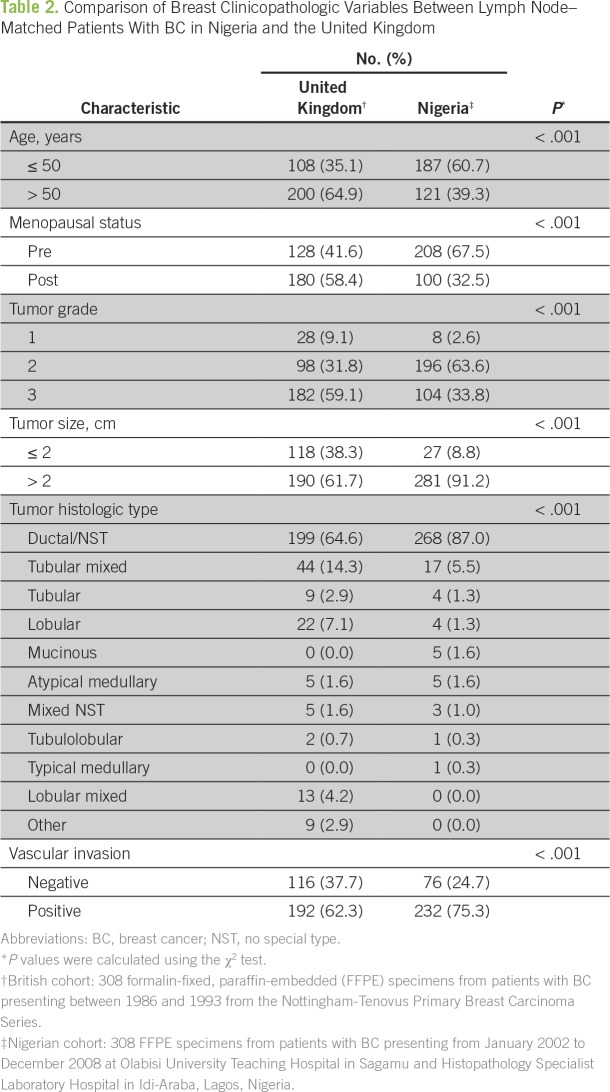
Comparison of Breast Clinicopathologic Variables Between Lymph Node–Matched Patients With BC in Nigeria and the United Kingdom

**Table 3:**
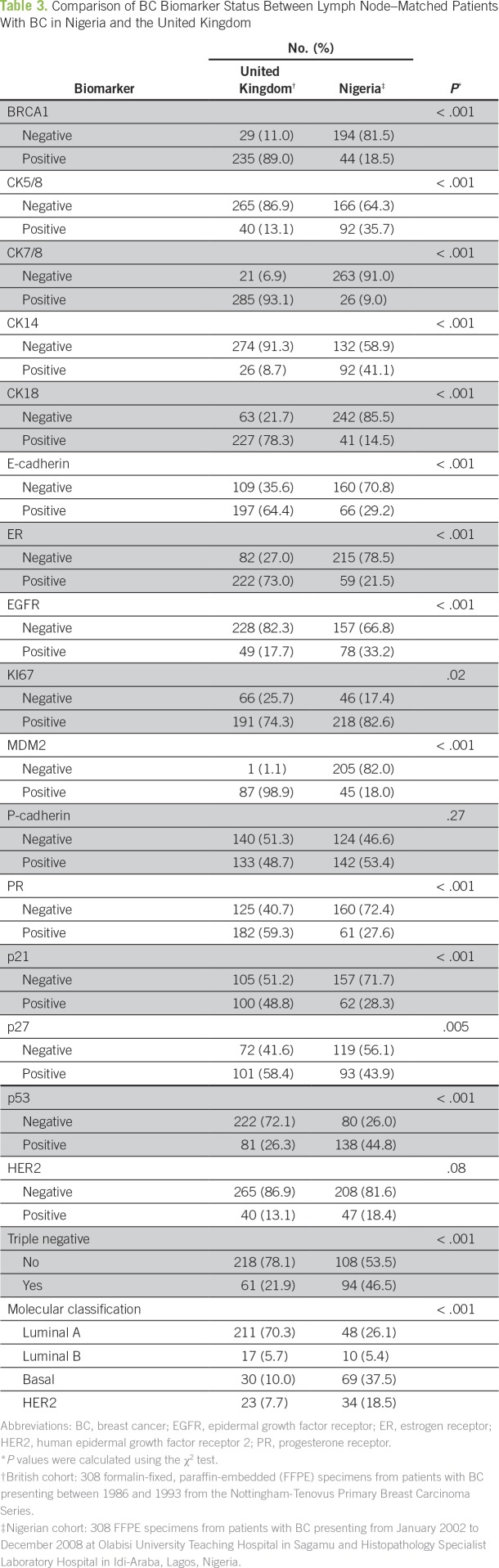
Comparison of BC Biomarker Status Between Lymph Node–Matched Patients With BC in Nigeria and the United Kingdom

## CHALLENGES OF TNBC: OPPORTUNITIES FOR GROWTH

**Fig 3:**
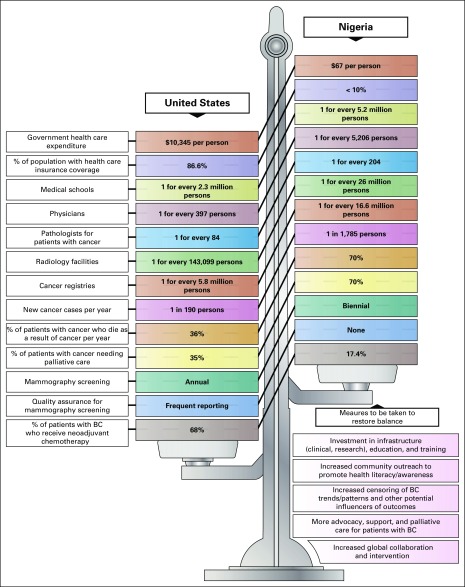
Comparison of health care infrastructure in Nigeria and the United States. BC, breast cancer.

This review spotlights TNBC in Nigeria, where the disease has reached crisis levels. Insights generated from underlying drivers of this high prevalence would affect our understanding of the etiology and biology of TNBC globally; guide research to improve clinical management of TNBC, especially in women of African ancestry; and help design evidence-based, patient-centered, holistic, and efficacious policy frameworks and health awareness programs that factor in resource constraints while targeting key risk factors and cultural issues that may be exacerbating the burden of TNBC in certain nations or ethnic groups. The stark gap in adequate cancer care between Nigeria and the United States and strategies to reduce this gap are shown in Figure 3. The challenges presented by TNBC worldwide and racial disparity in the United States require multilevel solutions and interventions, because the upstream determinants of outcomes span multiple domains of influence that are complexly intertwined. True impact will require targeted global initiatives like ICART, robust partnerships among governments and implementing agencies, judicious resource allocation, political will, incentives to promote innovation, team science, and focus on grassroots measures that have both the required reach and efficacy.

“Success is a staircase, not a doorway.” —Dottie Walters

## Figures and Tables

**Table A1:**
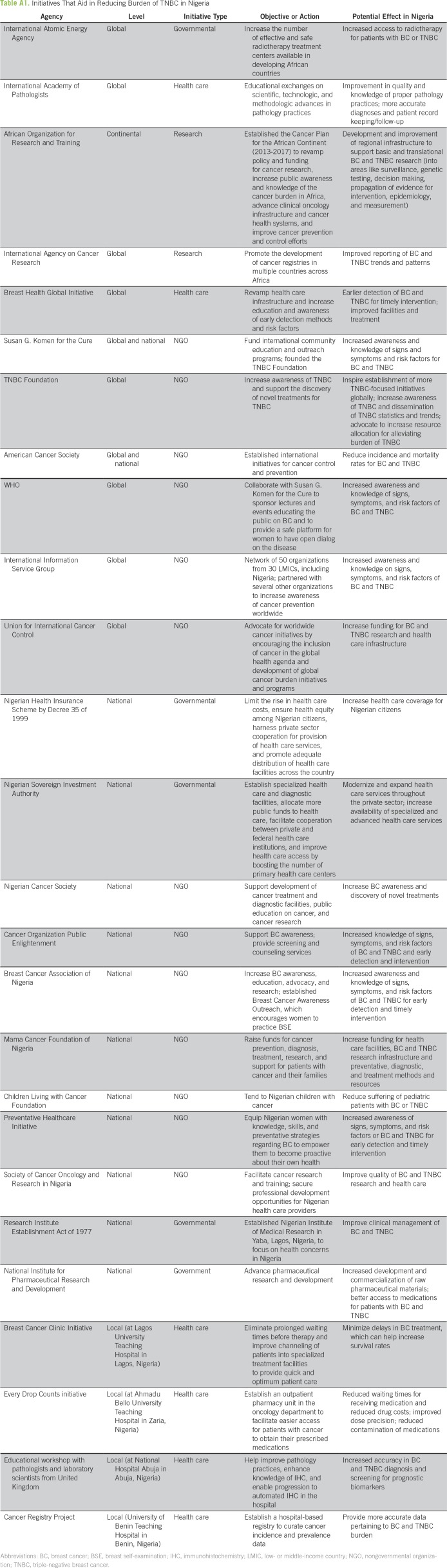
Initiatives That Aid in Reducing Burden of TNBC in Nigeria
